# Iliofemoral Venous Occlusion Due to Previous Intravenous Drug Abuse Crossed by Sharp Recanalization Technique With Endovascular Stenting: A Case Report

**DOI:** 10.1016/j.jscai.2026.104257

**Published:** 2026-02-24

**Authors:** Paul Segui, Valérie Monnin-Barès, Hamid Zarqane, Juliette Vanovershelde, Sébastien Bommart, Hélène Vernhet-Kovacsik

**Affiliations:** a Radiology Department, CHRU Montpellier, Montpellier, France; b INSERM U 1046, CNRS UMR 9214, PHYMEDEXP, Montpellier University, Montpellier, France

**Keywords:** case report, endovascular therapy, iliofemoral obstruction, postthrombotic syndrome, sharp recanalization, venous stenting

## Abstract

Recanalization of chronic venous occlusions is sometimes technically challenging. We present a rare case of venous occlusion, due to previous intravenous femoral drug use, with a fibrotic perivenous tissue in the groin, hard to cross. This patient presented with severe venous claudication with an active ulcer. After failure of conventional attempts, we opted for a more aggressive strategy using a modified Chiba needle (Cook Medical)–assisted sharp recanalization technique. We achieved initial technical success in recanalization, but thrombosis eventually recurred because of residual compression on the created extravascular tract. However, the patient showed clinical improvement, and we chose not to attempt another procedure. This case report emphasizes the use of this unconventional crossing technique and provides a detailed, didactic description of the equipment and procedural steps, aiming to illustrate its potential role and limitations in complex venous recanalization.

## Introduction

Endovascular treatment is currently the first-line approach in postthrombotic syndrome management.^1^, ^2^, ^3^, ^4^ However, recanalization of chronic venous occlusions is sometimes technically challenging. Sharp recanalization can help when obstructive lesions cannot be crossed with conventional techniques.^5^ We report the case of a hard fibrotic occlusion due to previous intravenous drug use, successfully crossed with a modified Chiba needle–assisted sharp recanalization technique.

## Background

Management of iliofemoral venous occlusions has evolved, with endovascular therapy becoming the mainstay. In chronic occlusions, angioplasty and dedicated venous stenting have shown favorable midterm outcomes, though restenosis and in-stent occlusion remain limitations. When conventional techniques fail, “sharp recanalization” techniques using specialized catheters or needles have been described as bailout strategies, albeit with increased technical complexity and potential risks.

Iliofemoral occlusions related to intravenous drug use are rarely reported but represent a challenging subgroup. Repeated femoral injections can induce extensive fibrosis and perivenous scarring, leading to chronic venous obstruction with disabling symptoms such as claudication and ulceration. This context underscores the need for alternative strategies in cases resistant to standard endovascular crossing.

## Case report

A 51-year-old man presented with postthrombotic syndrome of the left lower limb with an active venous ulcer and severe venous claudication with CIVIQ-20 score of 77 and Villalta score of 22.^6^^,^^7^ He had a history of deep vein thrombosis in 2010 in the context of intravenous drug and alcohol use, with femoral vein injection complicated by local infection in the groin area.

**Figure 1:**
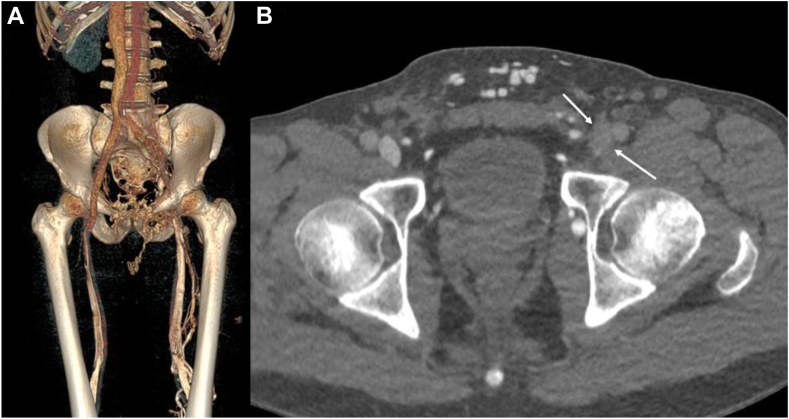
**Preprocedural imaging. (A)** Direct computed tomodensitometry venography with volume rendering technique reconstruction shows complete occlusion of the left common femoral vein in the projection of the femoral head, with postthrombotic stenosis of the iliac vein with a preserved femoropopliteal axis. **(B)** Axial native image shows fibrotic perivenous tissue in the groin (arrows), due to previous intravenous femoral drug use.

Direct CT venography revealed postthrombotic obstruction extending from the common femoral vein to the common iliac vein (Figure 1A).

**Figure 2:**
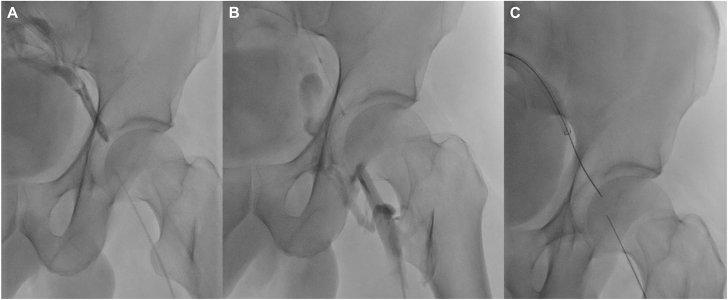
**The first endovascular procedure.** Venogram **(A, B)** confirms total occlusion of the left common femoral vein with failure of antegrade and retrograde attempts to cross it despite using the back end of the guide wire and guiding sheath **(C)**.

First, an endovascular procedure was scheduled under local anesthesia and sedation. The right internal jugular and left femoral vein were accessed by ultrasound-guided puncture. Retrograde recanalization was achieved to the level of the acetabulum using a stiff hydrophilic guide wire, but failed beyond it, despite using the back end of the guide wire and guiding sheath. Antegrade attempts were also unsuccessful and painful, with an extremely hard fibrous occlusion about 1 to 2 cm high in projection of the femoral head, which cannot be crossed (Figure 2).

Computed tomography venography review identified a perivenous fibrous thickening likely linked to prior intravenous drug use in the groin, corresponding to the area not crossed during the first recanalization (Figure 1B).

A multidisciplinary discussion led to a second attempt under general anesthesia 6 months later, as the patient was in a therapeutic impasse with disabling postthrombotic syndrome and an active ulcer.

**Figure 3:**
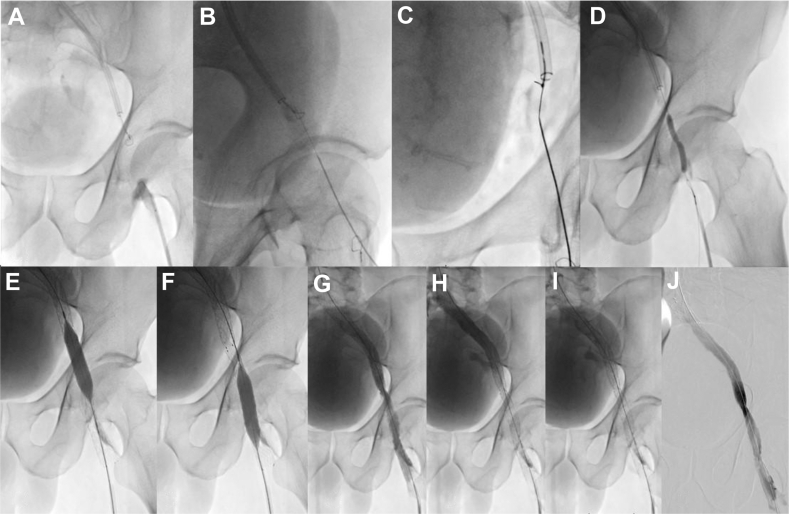
**The second endovascular procedure. (A)** Two 10-mm Amplatz Goose Neck snares are placed via each access upon contact with each venous stump above and below the fibrotic block. (**B**) The needle is gradually advanced under fluoroscopic guidance using different angles, endovenously as close as possible, crossing the occlusion. (**C**) The 0.018-inch guide wire inserted through the needle and upper snare is tightened over its tip. (**D**) Predilatation of the newly created tract with a 4 mm balloon. (**E-F**) Endovascular stenting and angioplasty. (**G-J**) Completion venography shows the restoration of flow with the disappearance of collaterals.

Again, we performed a double jugular and left femoral venous access. Inspired by Cupidon’s strike technique described in arterial recanalization,^8^ we placed two 10-mm Amplatz Goose Neck snares (EV3) via each access above and below the fibrotic block (after prior balloon angioplasty to widen the lumen of the upper venous stump to allow proper opening of the loop) (Figure 3). Under ultrasound guidance, we punctured the left femoral vein upstream from the lower snare with a 20G Chiba needle (88 mm). The needle was first inserted through the lower loop snare. Under fluoroscopic guidance using different angles, the needle was advanced endovenously as close as possible, crossing the occlusion. A 0.018-inch guide wire was inserted through the needle, and the upper snare was tightened over its tip. The tightened snare and the guide wire were moved en bloc cephalad up to the jugular sheath, placed through-and-through from the skin entry. The newly created tract was predilated using 4- and 5-mm balloons. Then, a 4F CXI support catheter (Cook) was introduced from the jugular approach up to the lower snare. A 0.014-inch guide wire was inserted in the catheter, and its soft tip was snared up to the femoral sheath with the 4F catheter. The 0.014-inch guide wire was then exchanged for a 0.035-inch stiff Amplatz wire, and we performed a balloon angioplasty with 9 mm and 12 mm high-pressure balloons (Conquest, Bard), with final breakthrough achieved at 30 atm. After an additional 12 mm balloon angioplasty on the iliac vein, we performed an ilio-femoral stenting with 2 overlapping 14 × 100 mm and 14 × 150 mm nitinol self-expanding stents (Optimed Sinusflex), dilated with 12- and 14-mm high-pressure balloons. Completion venography demonstrated the restoration of rapid flow with washing and disappearance of collaterals.

**Figure 4:**
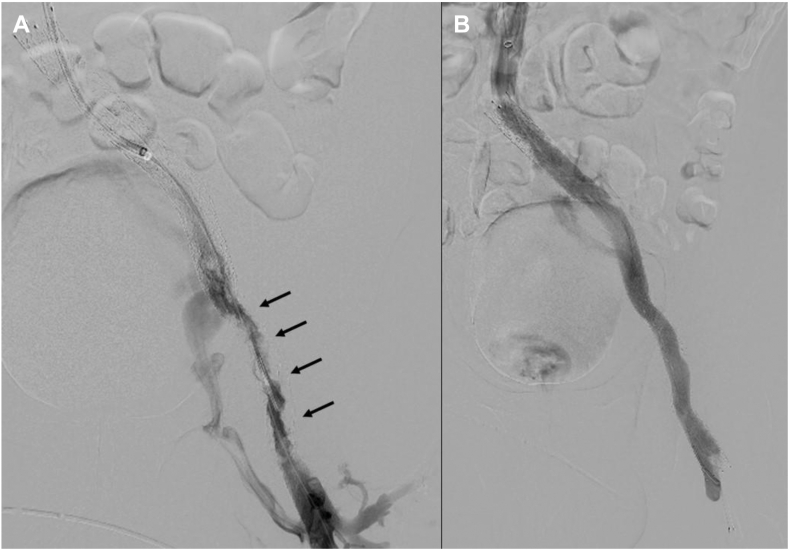
**The last endovascular procedure. (A)** Venography shows irregularity of the intrastent lumen, like a fatty protrusion through the stent mesh on the extravascular segment (black arrows). (**B**) Completion venography shows the restoration of stent patency and disappearance of collaterals.

An intravenous bolus of 50 UI/kg of unfractionated heparin was injected during each procedure. After recanalization, anticoagulation treatment included antiplatelet therapy and a therapeutic dose of low-molecular-weight heparin for the first month. The patient initially showed clinical improvement in both scores (Villalta from 22 to 10; CIVIQ from 72 to 29). Doppler at day 1 demonstrated patency of stenting, but the control at 2 weeks showed an occlusion confirmed by CT venography, also demonstrating an extravascular tract behind the fibrotic granuloma. A new intervention was planned 2 days later, with the same anticoagulation protocol as for the previous attempt (Figure 4). No material could be aspirated during the thrombectomy attempt. Venography showed irregularity of the intrastent lumen, like a fatty protrusion through the stent mesh on the extravascular segment. We decided to place a self-expandable covered stent (Viabahn, Gore) into the nitinol stent with an excellent angiographic result and stent patency on Doppler monitoring at day 1. However, thrombosis recurred 2 weeks later due to insufficient radial force of the covered stent with an aspect of plication of the Viabahn compressed by the fibrotic granuloma on CT venography. Further stenting with a nitinol device was considered, but the patient already showed partial improvement and was poorly cooperative. The medical staff, therefore, rejected the additional procedure. The patient was unfortunately lost to follow-up.

## Discussion

Sharp recanalization of occluded veins has been previously described, mainly for central venous recanalization.^8^^,^^9^ Most of the postthrombotic femoro-iliac occlusions can be crossed with conventional approaches, with the possible help of sheaths or triaxial catheter systems. Sharp recanalization techniques should be considered only after the failure of these techniques. Given their inherent risk of complication, the risk-benefit balance should always be considered by the operator.^10^

In this case, the uncrossable occlusion was due to a rock-solid fibrotic tissue of the groin due to previous drug injections in the femoral vein. Indeed, the chemical effect of injected drugs can lead to endothelial damage, thrombosis, and destruction of surrounding soft tissues.^11^

Chiba needle–assisted sharp recanalization is usually performed through a 4F sheath placed in the upstream vein. Because of the short and superficial nature of this occlusion and because of the venous curvature, we opted for a direct percutaneous puncture between 2 loop snares, placed via each access, enabling accurate orientation of the needle trajectory and successful passage through the rigid fibrotic occlusion. The use of a transseptal needle could have been discussed, with possible adjustable curvature, but a limitation of this device is the increased difficulty in penetrating heavily fibrotic occlusions.

A similar case recently reported in the literature^12^ demonstrated stent patency at the 3-month follow-up, but the authors did not specify whether a conventional recanalization attempt had been performed before the sharp recanalization technique, and the occlusion was shorter and located lower according to the iconography, probably easier to cross, with less angulation and, therefore, less risk of extravascular tract. Moreover, the occlusion was only diagnosed on sonography without additional imaging, which does not allow for assessment of peripheral tissues (fibrous granuloma complicating the crossing of the occlusion in our case).

We should have used a covered stent from the first procedure because of the partially extravascular tract, but we chose a nitinol self-expanding dedicated venous stent with an open-cell design for its radial strength. The best solution would probably have been to use a covered stent from the outset, strengthened by a nitinol stent inside. Procedural cone beam computed tomography images would have allowed us to better understand the recanalization path and, thus, guide the choice of stents.

## Conclusion

Sharp recanalization of challenging occlusions may require creative approaches. Despite the disappointing midterm patency outcomes associated with this procedure, this original approach initially made it possible to successfully cross an extremely hard occlusion related to drug crystals without complication. Sharp recanalization techniques should only be considered after failure of conventional approaches, keeping in mind the expected clinical benefit versus the risks inherent in such procedures.
